# EANM procedural guidelines for PET/CT quantitative myocardial perfusion imaging

**DOI:** 10.1007/s00259-020-05046-9

**Published:** 2020-11-02

**Authors:** Roberto Sciagrà, Mark Lubberink, Fabien Hyafil, Antti Saraste, Riemer H. J. A. Slart, Denis Agostini, Carmela Nappi, Panagiotis Georgoulias, Jan Bucerius, Christoph Rischpler, Hein J. Verberne, Cardiovascular Committee of the European Association of Nuclear Medicine (EANM)

**Affiliations:** 1 Nuclear Medicine Unit, Department of Experimental and Clinical Biomedical Sciences “Mario Serio”, University of Florence, Largo Brambilla 3, 50134 Florence, Italy; 2 Radiology & Molecular Imaging, Department of Surgical Sciences, Uppsala University, Uppsala, Sweden; 3 Medical Physics, Uppsala University Hospital, Uppsala, Sweden; 4 Department of Nuclear Medicine, DMU IMAGINA, APHP, Hôpital Européen Georges-Pompidou, University of Paris, Paris, France; 5 Université de Paris, PARCC, INSERM 690, Paris, France; 6 Turku PET Centre, Turku University Hospital, University of Turku, Turku, Finland; 7 Heart Center, Turku University Hospital, Turku, Finland; 8 Department of Nuclear Medicine and Molecular Imaging, University Medical Center Groningen, Groningen, The Netherlands; 9 Faculty of Science and Technology Biomedical, Photonic Imaging, University of Twente, Enschede, The Netherlands; 10 Department of Nuclear Medicine, CHU Cote de Nacre, Normandy University, EA 4650, Caen, France; 11 Department of Advanced Biomedical Sciences, University Federico II, Naples, Italy; 12 Department of Nuclear Medicine, Faculty of Medicine, University of Thessaly, University Hospital of Larissa, Larissa, Greece; 13 Department of Nuclear Medicine, University Medicine Göttingen, Georg-August University Göttingen, Göttingen, Germany; 14 Department of Nuclear Medicine, University Hospital Essen, University of Duisburg-Essen, Essen, Germany; 15 Department of Radiology and Nuclear Medicine, Amsterdam UMC, location AMC, University of Amsterdam, Amsterdam, The Netherlands

**Keywords:** PET, Myocardial blood flow, Myocardial flow reserve, Quantitative imaging

## Abstract

The use of cardiac PET, and in particular of quantitative myocardial perfusion PET, has been growing during the last years, because scanners are becoming widely available and because several studies have convincingly demonstrated the advantages of this imaging approach. Therefore, there is a need of determining the procedural modalities for performing high-quality studies and obtaining from this demanding technique the most in terms of both measurement reliability and clinical data. Although the field is rapidly evolving, with progresses in hardware and software, and the near perspective of new tracers, the EANM Cardiovascular Committee found it reasonable and useful to expose in an updated text the state of the art of quantitative myocardial perfusion PET, in order to establish an effective use of this modality and to help implementing it on a wider basis. Together with the many steps necessary for the correct execution of quantitative measurements, the importance of a multiparametric approach and of a comprehensive and clinically useful report have been stressed.

## Preamble

The European Association of Nuclear Medicine (EANM) is a professional non-profit medical association that facilitates communication worldwide among individuals pursuing clinical and research excellence in nuclear medicine. The EANM was founded in 1985. These guidelines are intended to assist practitioners in providing appropriate nuclear medicine care for patients. They are not inflexible rules or requirements of practice and are not intended, nor should they be used, to establish a legal standard of care. The ultimate judgement regarding the propriety of any specific procedure or course of action must be made by medical professionals taking into account the unique circumstances of each case. Thus, there is no implication that an approach differing from the guidelines, standing alone, is below the standard of care. To the contrary, a conscientious practitioner may responsibly adopt a course of action different from that set out in the guidelines when, in the reasonable judgement of the practitioner, such course of action is indicated by the condition of the patient, limitations of available resources or advances in knowledge or technology subsequent to publication of the guidelines. The practice of medicine involves not only the science but also the art of dealing with the prevention, diagnosis, alleviation and treatment of disease. The variety and complexity of human conditions make it impossible to always reach the most appropriate diagnosis or to predict with certainty a particular response to treatment. Therefore, it should be recognised that adherence to these guidelines will not ensure an accurate diagnosis or a successful outcome. All that should be expected is that the practitioner will follow a reasonable course of action based on current knowledge, available resources and the needs of the patient to deliver effective and safe medical care. The sole purpose of these guidelines is to assist practitioners in achieving this objective.

## Introduction—rationale

The use of PET (positron emission tomography) tracers for myocardial perfusion imaging (MPI) began in the 1980s [1]. Already in those very early years, together with the visual assessment of the uptake pattern, quantitative methods for measuring myocardial blood flow (MBF) were proposed and developed [2–5]. However, due to logistic constraints caused by the high costs of imaging systems and the limited availability of on-site cyclotrons needed for the production of then available perfusion tracers with short half-lives, myocardial perfusion PET was restricted to few research centres. Recently, the exponential growth of PET imaging in oncology has led to a major increase in the number of installed PET scanners. In addition, the availability of PET perfusion radiotracers that do not require an on-site cyclotron, such as (generator-based) Rubidium-82 (^82^Rb) or new Fluorine-18 (^18^F)-labelled radiotracers, is likely to increase in Europe in the coming years. Combined, these factors have increased the accessibility of PET MPI and have revived the interest in PET as a modality for the quantitative assessment of myocardial perfusion. Nevertheless, the acquisition and analysis of quantitative PET MPI is demanding and requires a high level of expertise.

The objectives of these guidelines are to promote the standardisation of acquisition protocols for quantitative PET MPI and propose up-to-date diagnostic criteria for the interpretation of PET MPI. In addition, these guidelines provide some insights into the clinical applications of quantitative PET MPI.

## PET technology

### PET imaging systems

**Fig. 1:**
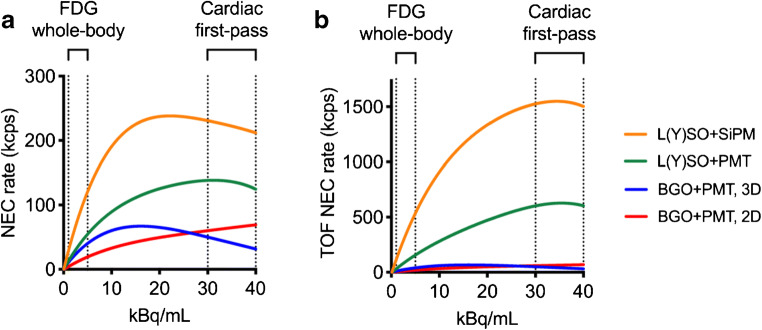
Typical noise-equivalent count (NEC) rate curves. BGO = bismuth germanate, GSO = gadolinium oxyorthosilicate, LSO = lutetium oxyorthosilicate, LYSO = lutetium-yttrium oxyorthosilicate, PMT = photomultiplier, SIPM = silicon photomultiplier. **a** NEC rates. **b** NEC rates accounting for image quality improvements due to time of flight. Typical count rate ranges during the first pass of a dynamic acquisition over the heart, as well as during a routine whole-body [^18^F]FDG scan, are indicated. **b** The advantage of modern LYSO+SiPM scanners during first-pass imaging compared with BGO systems is clearly shown

Dynamic cardiac PET is probably the most demanding protocol in terms of PET acquisition. During the first pass through the heart, the entire injected radioactivity is inside the field of view of the scanner, resulting in very high count rates. A few minutes later, both the short half-lives of especially Oxygen-15 (^15^O) and ^82^Rb and the distribution of the radioactivity throughout the entire body result in very low count rates. To put this in perspective, the amount or radioactivity within the field of view of the scanner is 20–40 times higher during the first pass in a dynamic scan than during a typical whole-body [^18^F]-fluorodeoxyglucose ([^18^F]FDG) scan, whereas at the end of the dynamic scan, it is about a factor 100 lower, approximately between 25 and 50% of that in a whole-body [^18^F]FDG scan. Figure 1 illustrates the noise-equivalent count (NEC) rate capabilities of different types of PET scanners, the properties of which will be discussed in further detail in the paragraphs below.

#### 2D vs. 3D

In a 2-dimensional (2D) PET scanner, detector rings are separated by septa (e.g. lead or tungsten rings). These septa partially shield coincidences from occurring between detectors in one ring and detectors in a non-adjacent or more distant rings and will reduce scattered events. A scanner without septa is referred to as a 3-dimensional (3D) scanner. This 3D mode allows for coincidences between all available rings, significantly increasing sensitivity and count rate per detector, but on the other hand increasing scatter and randoms as well. However, the advantages due to improved sensitivity far outweigh the disadvantages due to increased scatter and randoms (Fig. 1). The latest generations of PET/computed tomography (CT) scanners are no longer equipped with septa, because improved detector technology and faster electronics allow for better handling of high count rates, and increased computing power and current reconstruction algorithms allow to deal with the much larger amounts of raw data produced in 3D PET [6].

#### Crystals

The most common crystal types applied in PET are BGO (bismuth germanate), GSO (gadolinium oxyorthosilicate), and the lutetium-based crystals, such as LSO (lutetium oxyorthosilicate) and LYSO (lutetium-yttrium oxyorthosilicate). Each type of crystal has been used for cardiac imaging. Table 1 gives an overview of some important properties of these crystals. Using BGO, NEC rates are higher in 2D mode than in 3D mode during the first pass of PET tracer during a dynamic cardiac scan (Fig. 1a). This is the reason why cardiac PET was preferably done in 2D mode on older scanners. As also seen in Table 1 and Fig. 1a, LSO and LYSO detectors, because of their shorter light decay time combined with fast electronics, allow for higher count rates, resulting in much higher 3D mode NEC rates during cardiac first-pass imaging than BGO.

**Table 1:** Properties of commonly used crystals for PET scanners

	BGO	GSO	LSO	LYSO
Density (g/cm^3^)	7.1	6.7	7.4	7.4
Attenuation length (mm)	10.4	14.1	11.4	11.8
Light output (photons/MeV)	9000	8000	30,000	30,000
Light decay time (ns)	300	60	40	40

*BGO* = bismuth germanate, *GSO* = gadolinium oxyorthosilicate, *LSO* = lutetium oxyorthosilicate, *LYSO* = lutetium-yttrium oxyorthosilicate

#### Time-of-flight

Time-of-flight (TOF) capability can increase the signal to noise ratio of the images [7, 8]. Figure 1b shows NEC rates taking the gain due to TOF into account as well, but this is valid only for the NEMA NEC measurement with a 20-cm phantom [9, 10]. TOF benefits in a clinical cardiac scan are lower than what is shown here, but also in a clinical situation TOF will result in a considerable increase in signal to noise ratio compared with non-TOF reconstructions [11]. In this regard, TOF can improve image quality in cardiac perfusion imaging [12].

#### Solid-state scanners

Until recently, most PET systems were equipped with photomultiplier tubes (PMT) to convert the light from the scintillating crystals into an electronic pulse. The advent of PET-magnetic resonance (MR) mandated the development of new technologies, since PMTs do not work well in a magnetic field. In the latest generation of PET scanners, PMTs have been replaced by silicon photomultipliers (SiPM), either in a traditional block detector configuration, coupling an array of crystals to a smaller number of SiPMs, or coupled one-to-one to scintillating crystals (‘direct photon counting’). Digital PET systems allow for better TOF resolution and improved sensitivity (in case of block configurations), further improving image quality [13–15]. This is shown in Fig. 1b, stressing the advantages of last generation PET systems with SiPMs and high-efficiency TOF in terms of count rate performance during first pass in dynamic scans. So far, there are no available data about the specific advantages for quantitative cardiac PET. However, computation of parametric MBF images, showing MBF rather than just tracer uptake at the voxel level, requires good count statistics, and thus, the digital scanners with their improved count rate capabilities are helpful.

## Radiopharmaceuticals for myocardial perfusion imaging with PET (Table 2)

### [^15^O]water

**Fig. 2:**
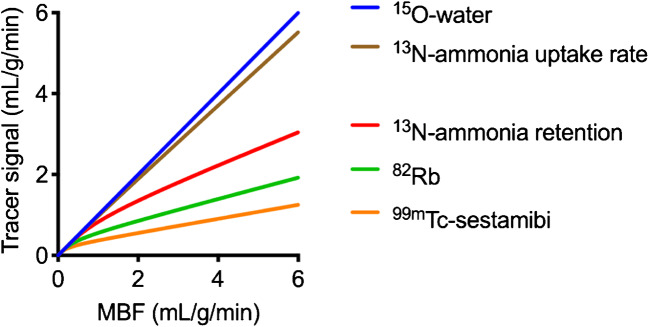
Transport rate constant from plasma to tissue (*K*_1_) as function of MBF for [^15^O]water, [^13^N]NH_3_ [2] and ^82^Rb [29] compared with the SPECT tracer [^99m^Tc]Tc-sestamibi [30]. For [^13^N]NH_3_, curves based on uptake rate (*K*_1_) and on retention, that is, the transport rate into the metabolically trapped compartment, are given

[^15^O]water PET is considered the reference for non-invasive in vivo measurement of MBF because it is metabolically inert and essentially freely diffusible and has an extraction fraction close to one up to very high flow values (Fig. 2) [3, 18]. The 122 s radioactive half-life of ^15^O implies that [^15^O]water can only be used at hospitals with an on-site cyclotron.^15^O is produced either by irradiating enriched Nitrogen-15 (^15^N) with protons, using the ^15^N(p,n)^15^O reaction, or by irradiating natural nitrogen with deuterons using the ^14^N(d,n)^15^O reaction. The advantage of the latter production method is that the target material is basically air and, hence, cheap and that the required deuteron energy is only 3 MeV, allowing for construction of small, dedicated cyclotrons requiring limited shielding, at a considerably lower cost than regular PET cyclotrons [19]. These cyclotrons have until now only been installed at a few mainly research hospitals, but their wider spread could facilitate an increased clinical use of [^15^O]water. Using the (p,n) reaction allows for production of [^15^O]water on standard medical cyclotrons. The maximum positron energy of ^15^O of 1.7 MeV is higher than that of ^13^N, but considerably lower than for ^82^Rb, leading to a spatial resolution somewhere in between that of ^13^N and ^82^Rb [20]. In the setting of high-resolution PET scanners (FWHM = 3 mm), the resolution loss could be estimated to be around 0.5 mm [21]. Although the properties of [^15^O]water imply that perfusion can be measured accurately irrespective of metabolic status, a challenge associated with its freely diffusible nature is that the tracer is not retained in the myocardium. No static uptake images can be acquired to give an initial, qualitative image indicating perfusion defects or myocardial viability. Tracer kinetic modelling is required to get absolute perfusion values, and meaningful perfusion images can only be obtained by performing this modelling on a voxel level. On the other hand, the free diffusibility of [^15^O]water allowed for the introduction of the perfusable tissue fraction (PTF) concept, an intrinsic partial volume correction, the mathematical details of which are described in the “Quantification of myocardial blood flow”, [^15^O]water” section [22]. This is a major difference with other perfusion agents: MBF measurements based on ^82^Rb or [^13^N]ammonia ([^13^N]NH_3_), or any other tracer except [^15^O]water, suffer from partial volume effects due to the limited spatial resolution of PET, whereas [^15^O]water measurements do not. Furthermore, the PTF can be used to estimate the so-called perfusable tissue index (PTI) which has been shown to be a marker of tissue viability [23]. As such, perfusion and viability, distinguishing ischaemia from infarction, can be measured using a single scan. Tracer kinetic analysis of [^15^O]water used to be time-consuming, but recent years have seen the development of a number of software packages that nearly automatically supply MBF values based on dynamic scans, or are even capable of automated calculation of parametric images of MBF, PTF, and blood volume, and even left ventricle (LV) volumes and ejection fraction (EF) based on either first-pass gated images or gated parametric blood volume images [16, 17]. The short half-life of [^15^O]water allows for the completion of a rest–stress protocol within 30 min.

**Table 2:** Comparison of the available tracers for quantitative perfusion PET

	[^15^O]water	[^13^N]NH_3_	^82^Rb	[^18^F]flurpiridaz
Radionuclide half-life	122 s	9.96 min	75 s	109.8 min
Availability	On-site cyclotron	On-site cyclotron	Generator	Cyclotron (possible shipping)
Mean positron range in water (mm) [20]	2.5	1.5	5.9	0.6
Relationship with MBF	Ideal (freely diffusible)	High extraction fraction	Non-linear extraction fraction	High extraction fraction
Image quality	Parametric MBF images only	Good to high	Fair to good	Very high
Gated imaging	Possible from first pass (blood pool)	High quality	Good quality	High quality
Time schedule	Very tight	Interval between rest and stress injections	Very tight	Separate stress and rest injections
Previous experience	Used mainly in research setting and with hybrid imaging	Widely used qualitatively and quantitatively	Widely used qualitatively and quantitatively	Presently ongoing phase III trial

*MBF* = myocardial blood flow

#### Dosimetry

[^15^O]water: 1 mSv/GBq [24–26]. A typical protocol consisting of two injections of 400 MBq will result in an effective dose of 0.8 mSv.

### ^82^Rb

^82^Rb has been employed as a myocardial perfusion agent since the early 1980s and has been extensively used for qualitative and quantitative cardiac PET [5, 27–29]. ^82^Rb decays with a half-life of 75 s with emission of a positron with a maximal energy of 3.15 MeV in 95.5% of decays or by electronic capture in 4.5% of decays with subsequent gamma emission. The high energy range of these positrons results in an intrinsic lower spatial resolution of PET images than with [^13^N]NH_3_ or ^18^F-radiolabelled tracers [20]. In particular, the resolution loss in soft tissues has been calculated to be greater than 2 mm [21]. ^82^Rb has the same biological properties as potassium (K^+^) and is extracted by the myocardium through the Na-K-ATPase pump. ^82^Rb is taken up in viable myocardium, whilst it is rapidly cleared from fibrotic tissue. The extraction of ^82^Rb during the first pass is high (65%) but decreases in a non-linear way with increasing blood flow [28, 29]. This effect is clear when compared with [^13^N]NH_3_ or [^15^O]water, although extraction is slightly better than for the most common ^99m^Tc-labelled radiopharmaceuticals [30] (Fig. 2). ^82^Rb can be eluted on demand from a Strontium-82 (^82^Sr)/^82^Rb generator. In 1986, Gould et al. published the first clinical use of an ^82^Sr/^82^Rb generator for the detection of coronary artery disease (CAD) [31]. ^82^Rb PET demonstrated higher diagnostic performance in comparison with single-photon emission computed tomography (SPECT) with ^201^Thallium (^201^Tl), and this supported the approval by the Food and Drug Administration (FDA) of ^82^Sr/^82^Rb generators for clinical use in the USA [32]. The ^82^Sr/^82^Rb generators are commercially available in the USA and Canada and are currently in the process of approval for clinical use in Europe. The ^82^Sr/^82^Rb generator is positioned into a dedicated injection system that flushes saline through the generator. Volume and flow rate of saline can be manually selected and adapted to the ‘age’ of the generator. ^82^Rb is eluted from the generator with a volume of saline between 10 and 50 mL and at a flow rate between 20 mL/min at the beginning of the life of the generator up to 35 mL/min at the end of the life of the generator because the concentration of ^82^Rb decreases over time in the eluate. Recommended activities of ^82^Rb to inject to patients are 10 MBq/kg (with a minimal dose of 740 MBq and maximal dose of 1480 MBq) for PET acquisitions in a 3D mode. However, generators may need re-calibration when administered activity is changed, which makes weight-based dosing impractical in high-throughput centres. Furthermore, the high end of this interval may increase the risk of detector saturation. The use of fixed doses ranging from 740 to 1110 MBq, according to the PET/CT device sensitivity, is as well acceptable. As these injected activities should be doubled for PET acquisitions in 2D mode, we advocate the use of 3D mode acquisitions. One important concern regarding ^82^Sr/^82^Rb generators is the risk of ^82^Sr and ^85^Sr breakthrough. This risk increases with the ‘age’ of the generator and the total volume of eluate. The level of ^82^Sr and ^85^Sr in the eluate should be monitored daily. It can be estimated by measuring the residual activity in the eluate after complete decay of ^82^Rb. Newer ^82^Sr/^82^Rb generator and injection systems have the advantage of providing automated controls of residual ^82^Sr and ^85^Sr activities in the eluate. In addition, these systems are equipped with a second injector that is connected to the infusion system at the exit of the ^82^Sr/^82^Rb generator. This second injector allows for a constant administration rate of ^82^Rb independent of the “age” of ^82^Sr/^82^Rb generator and the concentration of ^82^Rb in the eluate. ^82^Rb is well suited for clinical use of PET MPI. First of all, ^82^Rb can be obtained on demand after elution of the ^82^Sr/^82^Rb generator. Second, the short half-life makes sequential rest and stress PET acquisition possible in 30 min without the presence of residual activity in the myocardium of the first ^82^Rb injection. Finally, ^82^Rb accumulates in the myocardium allowing for the evaluation of viability and myocardial contractility. The intrinsic limitations of ^82^Rb are, however, the high energy of the emitted positron that worsens spatial resolution and thus the MPI quality, and the non-linear myocardial uptake at high blood flow that limits the precision of the quantification of hyperaemic/stress MBF.

#### Dosimetry

^82^Rb: 1.1 mSv/GBq [26, 33]. The effective dose depends on the exact protocol used, but two injections of 10 MBq/kg result in an effective dose of approximately 1.5 mSv.

### [^13^N]NH_3_

[^13^N]NH_3_ has been employed as myocardial perfusion agent since the seventies of the last century [34]. It is produced by a cyclotron by means of the ^16^O(p,α)^13^N reaction. It has a relatively low positron energy (1.19 MeV), with low-resolution loss (about 0.2 mm) [20, 21], and a 9.96-min half-life, which permit to acquire higher quality images than with the other commonly used tracers, although a rest–stress study requires a slightly longer acquisition time than with [^15^O]water and ^82^Rb [35]. In blood, [^13^N]NH_3_ is mainly present as ammonium ion (NH_4_^+^), which can cross the cell membrane through the sodium–potassium exchange system, whilst [^13^N]NH_3_ diffuses passively because of its lipophilicity. Within the cell, [^13^N]NH_3_ may enter various metabolic pathways, among which the glutamic acid–glutamine is the most important, or back diffuse to blood [36]. Thus, the final myocardial uptake is influenced by several variables, including flow, extraction fraction and metabolic status. It has been demonstrated that [^13^N]NH_3_ extraction is inversely and non-linearly related to blood flow, with values ranging from 0.8 at baseline flow to 0.6 at flow about 3 mL/min/g [35]. On the other hand, the metabolic effects are probably small [36]. [^13^N]NH_3_ can be effectively used for the evaluation of relative myocardial uptake, and it has been demonstrated to be superior to SPECT MPI in terms of sensitivity and specificity [37]. ECG-gated PET studies are of high quality, but the stress acquisition is performed with some delay after tracer injection, although much closer as compared with gated SPECT, and then might not represent the truly functional status during stress. Finally, and most importantly, [^13^N]NH_3_ is highly valuable for the absolute quantitative measurement of MBF [2, 4, 38–40].

#### Dosimetry

[^13^N]NH_3_: 2 mSv/GBq [26, 33]. A typical protocol consisting of two injections of 400 MBq will result in an effective dose lower than 1.8 mSv.

## PET acquisition protocols

### Stress protocols

**Fig. 3:**
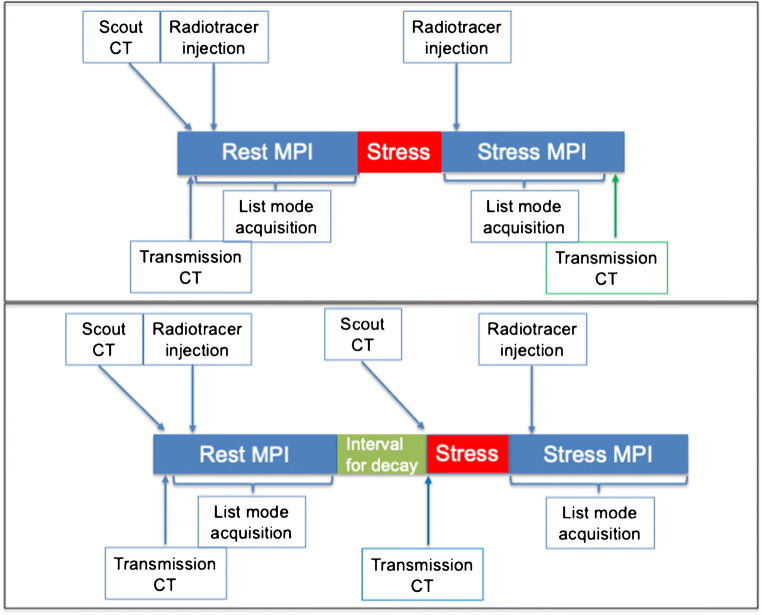
Protocols for rest–stress quantitative cardiac PET. The upper panel shows the sequence for tracers with short half-life ([^15^O]water, ^82^Rb). The lower panel shows the standard sequence for longer half-life tracers ([^13^N]NH_3_, [^18^F]flurpiridaz); however, using correction for residual activity, the shorter protocol can be adopted also for [^13^N]NH_3_

Because the quantitative MBF measurement requires the acquisition of the input function, pharmacologic stress is the sole possible option. The stress test modalities do not differ for the various tracers and are the same as for SPECT MPI [41], although the execution of the stress injection with the patient already positioned on the camera bed, together with the additional problem to avoid his/her motion, requires particular cautiousness (Fig. 3). The commonly used stressors are dipyridamole, adenosine and, most recently, regadenoson. Dipyridamole, however, is not approved for this indication in many European countries and cannot be recommended anymore. The commonly used vasodilators can be contraindicated in case of severe chronic obstructive pulmonary disease, in particular with asthma components, and an alternative stress test could be dobutamine combined with atropine, although it is still debated whether this allows for reaching maximal hyperaemia [42, 43]. Patients must be fasting for at least 6 h and refrain from caffeinated beverages, food and analgesics containing caffeine for at least 12 h and from xanthine containing drugs for 48 h. Dipyridamole/Persantin should be stopped 24 h prior to vasodilator infusion. Withdrawal of cardiac drugs can be considered according to the exam indications and the patient conditions. It is recommended to monitor arterial blood pressure and to record a 12-lead ECG during stress to identify ischaemic ECG changes and potential induced arrhythmias. It is advisable to thoroughly instruct patients on the necessity to remain relaxed and avoid movements even in case of symptoms. For all these circumstances, the use of regadenoson appears most advantageous in the setting of perfusion PET, both because of the more favourable symptom and adverse effect profile and because it significantly shortens and simplifies the stress protocol, reducing the degree of patient motion [44–46]. Moreover, similar values of stress MBF have been reported in the comparison with dipyridamole [47]. The best position is supine with the arms over the head. Because this position must be kept for a relatively prolonged time, all possible care must be given to make the patient comfortable and capable of remaining motionless without unduly effort. Arm rests or other supportive measures may be useful to this purpose.

### Acquisition protocols

In the majority of systems, CT for attenuation correction is acquired before the PET acquisition. Patients must be instructed to breathe regularly during CT acquisition (see “Attenuation correction” section) and to avoid any movement during the bed translation under the PET detector. A respiration-averaged low-dose CT can be acquired, using essentially a respiratory gating protocol with all gates summed. This provides an attenuation map that is the closest match to the PET images and, in this regard, should be considered the first choice, if feasible. However, a respiration-averaged CT covering the whole breathing cycle can result in a quite high radiation dose, so a free breathing, relatively slow CT is a good second-best option. The radiation dose of performing a low-dose CT in a patient is < 1 mSv, down to 0.1 mSv for an ultra-low-dose CT on a latest generation PET/CT scanner. The standard sequence for myocardial perfusion PET is rest–stress, since both studies are performed during the same session, and thus, the residual effects of the pharmaceutical stress agent on perfusion could affect the resting images. However, particularly using [^15^O]water in the setting of hybrid imaging including coronary CT angiography (CCTA), the acquisition of a sole stress study has been effectively performed (see “Hybrid imaging” section).

#### [^15^O]water

**Fig. 4:**
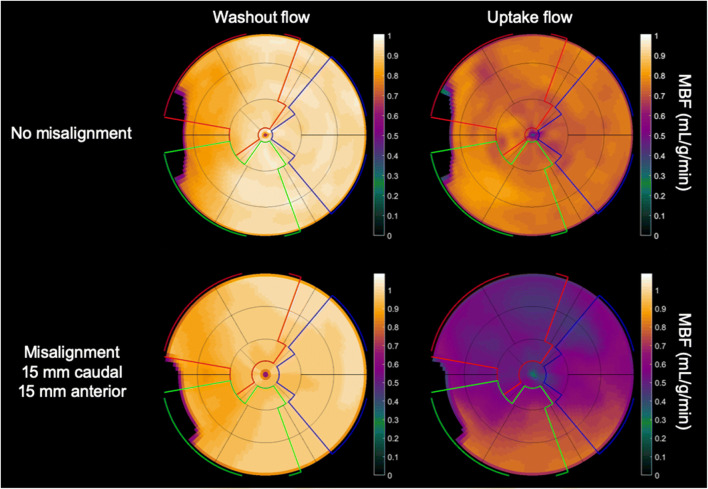
Effect of 15 mm PET/CT misalignment on absolute MBF for values measured from washout rate ([^15^O]water; left) and values measured from uptake rate (^82^Rb or [^13^N]NH_3_; right). Polar maps are based on the same simulated MBF scans for both cases. Misalignment results in a very slight increase in measured MBF for [^15^O]water and in a large anterior defect for ^82^Rb or [^13^N]NH_3_

In contrast to tracers that stay confined to the myocardium, [^15^O]water is freely diffusible and acquisition protocols are focused in all cases on obtaining arterial input function and tissue response to quantify MBF in absolute terms. For stress acquisition, the tracer is administered after maximum vasodilation has been achieved. [^15^O]water is then injected as a bolus followed by a saline flush, in case of adenosine preferably over a second intravenous access not to interfere with its continuous flow rate. Preferably, administration should be done using a fast-controlled automated injection to ensure a constant bolus, for example injection 5 mL of [^15^O]water at 1 mL/s followed by 35 mL saline at 2 mL/s [25]. A dynamic frame sequence is initiated upon injection of [^15^O]water with a duration of 4 min, which is sufficient since equilibrium between blood and tissue has been reached before that time point. It is essential to have a clear communication between the person on the infusion system and the technologist in the control room. Stress acquisition can be acquired as little as 10 min after the rest acquisition, given the short physical half-life of [^15^O]water. Due to the relative short biological half-life of adenosine, it is important that adenosine infusion is continued throughout the entire stress scan, which is another reason not to scan longer than 4 min. Respiration-averaged low-dose CT for attenuation correction is ideally obtained separately for rest and for stress [^15^O]water acquisitions to account for the anatomical different position of the heart during stress as compared with rest (i.e. ‘myocardial creep’). It has to be underscored that [^15^O]water modelling for quantification of perfusion is based on its clearance rate rather than uptake rate (see “Quantification of myocardial blood flow” section below). Since attenuation correction affects the amplitude of time–activity curves but does not further change the shape of these curves, an erroneous attenuation correction does not affect MBF values for [^15^O]water to a large extent (Fig. 4). Studies without attenuation correction have been conducted for [^15^O]water and have shown little impact on MBF values [25].

#### ^82^Rb

Acquisition usually starts with rest images to reduce the impact of residual effects of stress (myocardial stunning after ischaemia). As for [^15^O]water, low-dose CT acquisitions should be preferably obtained before the rest and after the stress for attenuation correction of PET images. Depending on the PET-CT system and patient position, it might be necessary to connect the infusion system of ^82^Rb to the patient only after the acquisition of the CT in order to keep the length of the infusion system as short as possible. In addition, acquisition protocols that use the low-dose CT acquired after the stress for attenuation correction for both rest and stress PET acquisitions have been proposed to reduce the radiation exposure of patients, but possible problems related to the ‘myocardial creep’ should be considered. A minimal duration of 10 min is requested between rest and stress injections so that the generator is fully replenished. The same protocol is followed for both rest and stress PET acquisitions. PET data are acquired in list mode (to allow re-binning for gated and dynamic datasets for MBF calculations) for at least 5 min. The PET acquisition should be ready to initiate on the system when the button to start the elution of the ^82^Sr/^82^Rb generators is pressed, and this should be clearly communicated. Then, the acquisition begins as soon as activity is detected on the PET detectors.

#### [^13^N]NH_3_

In case of [^13^N]NH_3_, at least 5 half-lives should be left between the two studies (i.e. ≈50 min). To optimise the patient throughput it is reasonable to proceed with the acquisition of the resting study of at least another patient and then come back to the former one for the stress acquisition. Shortened protocols for [^13^N]NH_3_ have been proposed, adjusting for residual activity of the resting injected dosage, but the effectiveness of the correction methods is not yet definitively established [48, 49]. For [^13^N]NH_3_, PET acquisitions are the same for the rest and the stress studies. Since patients have to be repositioned on the examination bed for the stress study, a second CT for attenuation is usually required. Tracer injection is ideally performed using an automated injection system and image acquisition is started immediately or a few seconds before the start of tracer injection. Again, clear communication between the involved personnel is mandatory. The optimal acquisition protocol is in list mode for approximately 10 min. The data will be subsequently re-binned to obtain the dynamic image sequence necessary for MBF measurement and gated images of the final tracer uptake for visual assessment and volume calculations. Alternatively, a list mode (or predefined frame mode) acquisition of about 10 min can be performed for quantitative MBF measurement, immediately followed by a single static rest and stress gated acquisition for another 5 to 10 min.

## Image reconstruction

### Recommendations common to all perfusion radiotracers

The acquired data are corrected for geometry, randoms, scatter, normalisation and dead time losses. Specific problems may arise in older scanners when the high activities injected may cause overflow and dead time problems especially when using ^82^Rb. The general recommendation is to use a pixel size of 2–3 mm, but slightly larger dimensions can be employed without affecting quantitation. Iterative reconstruction methods are nowadays the standard in most scanners performing 3D imaging. In general, these algorithms improve both image quality and the signal to noise ratio as compared with the standard filtered back projection, but there are still uncertainties on the best possible methodology, which also depends on the particularities of the individual scanner [50]. Furthermore, it must be considered that changing the type of reconstruction algorithm could influence the final MBF measurement [51, 52].

#### Attenuation correction

**Fig. 5:**
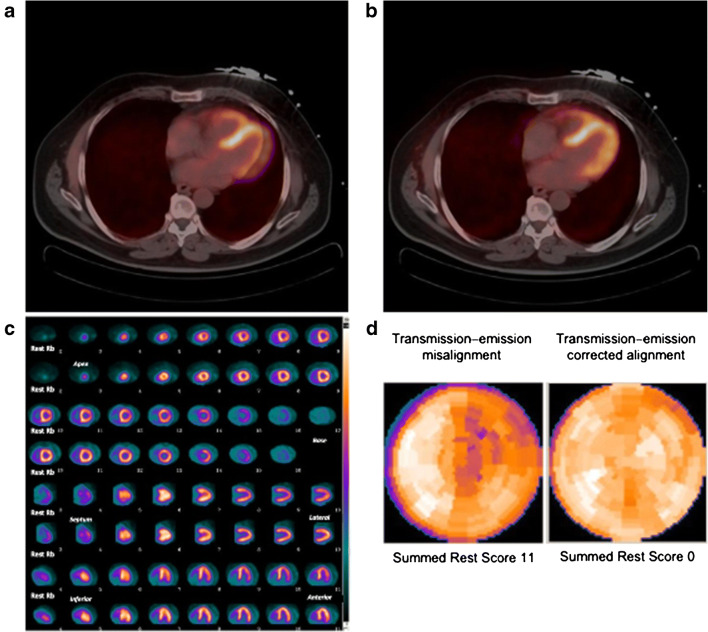
Transmission–emission misalignment example. Misalignment between CT transmission and rest ^82^Rb perfusion PET images (**a**) with correction of transmission–emission misalignment (**b**). Anterolateral perfusion defect on rest ^82^Rb perfusion images (**c**, upper rows) deriving from applying the incorrect attenuation coefficients during tomographic reconstruction to an area of LV myocardium overlying lung field on CT transmission scan, and normal rest perfusion study (**c**, lower rows) after correction, with relative polar maps (**d**)

In theory, older scanners with line-source-based attenuation correction are still valid for cardiac studies, because these transmission images are usually well comparable with the PET emission images. Indeed, the exact overlap between a high-resolution frozen image such as the CT acquired for attenuation correction and the blurred PET images, which are the sum of multiple heart cycles and breathing phases, remains a central problem for cardiac PET. Preferably, a respiration-averaged low-dose CT, using a protocol similar to that used for retrospective respiratory gating, should be used [53]. If this option is not feasible, a continuous shallow breathing (the same condition that the patient should be instructed to keep for the whole duration of the PET study, see above) is the preferred condition for achieving a CT image that can most effectively overlap with the PET image. However, images should always be checked for misalignment between PET and CT, and misalignment should be corrected for prior to reconstruction (Fig. 5) [54]. Metal artefacts can present a challenge for the reconstruction algorithms and must be compensated for to produce accurate attenuation maps. Currently, several metal artefact reduction methods have been introduced in modern CT systems [55].

#### Respiratory and patient motion correction

The adverse influence of breathing and patient motion on cardiac images has been well demonstrated. Several approaches have been proposed for correcting the respiratory and patient motion artefacts using respiratory gating, including motion estimates in the reconstruction algorithm and even by limiting the respiratory motion with an abdominal belt [56–58]. So far, however, none of these quite complex methods has been widely applied.

#### Dynamic images

The frame duration during the first pass of the radioactivity through the heart is usually no less than 5 s. After this, frame duration can be increased incrementally. Table 3 summarises some employed re-binning schemes for the various radiotracers. The sequence of the dynamic images on which the measurement of MBF will be performed must be checked for possible movement artefacts. Especially important is inter-frame motion, which is difficult to correct, but can heavily affect the accuracy of quantitative measurements, particularly during the rapid image sequence needed to obtain the time activity curves of the input function and of the initial tracer uptake. Even relatively limited breathing or patient movement can affect the position of the LV and of the myocardial wall. Some processing software packages have the capability to perform a motion correction. If this is not possible, the deletion of single frames could be considered.

**Table 3:** Examples of framing schemes for re-binning of dynamic list mode acquisitions

Reference	Tracer	Frame sequence	Total time
Kajander S et al. [59]	[^15^O]water	14 × 5 s; 3 × 10 s; 3 × 20 s; 4 × 30 s	4 min 40 s
Danad I et al. [60]	[^15^O]water	1 × 10 s; 8 × 5 s; 4 × 10 s; 2 × 15 s; 3 × 20s; 2 × 30 s; 2 × 60 s	6 min
Clinical protocol in Aarhus, Amsterdam, Uppsala	[^15^O]water	1 × 10 s; 8 × 5 s; 4 × 10 s; 2 × 15 s; 3 × 20s; 2 × 30 s	4 min
Muzik O et al. [38]	[^13^N]NH_3_	12 × 10 s; 4 × 15 s; 4 × 30 s; 3 × 300 s	20 min
Hutchins GD et al. [40]	[^13^N]NH_3_	12 × 10 s; 4 × 30 s; 1 × 360 s	10 min
DeGrado TR et al. [39]	[^13^N]NH_3_	12 × 10 s; 4 × 30 s; 3 × 120 s; 2 × 300 s	20 min
Sciagrà R et al. [61]	[^13^N]NH_3_	24 × 5 s; 2 × 30 s; 1 × 60; 1 × 300 s	9 min
El Fahkri et al. [62]	^82^Rb	24 × 5 s; 86 × 30 s	6 min
Lortie et al. [63]	^82^Rb	12 × 10 s; 2 × 30 s; 1 × 60 s; 1 × 120 s, 1 × 240 s	10 min
Dekemp RA et al. [64]	^82^Rb	9 × 10 s; 3 × 30 s; 1 × 60 s; 1 × 120 s	6 min
Dekemp RA et al. [64]	^82^Rb	12 × 10 s; 2 × 30 s; 1 × 60 s; 1 × 120 s	6 min
Dekemp RA et al. [64]	^82^Rb	12 × 5 s; 6 × 10 s; 4 × 20 s; 4 × 40 s	6 min
Armstrong IS et al. [52]	^82^Rb	1 × 10 s; 8 × 5 s; 3 × 10 s; 2 × 20 s; 4 × 60 s	6 min
Gaudieri V et al. [65]	^82^Rb	12 × 5 s; 6 × 10 s; 4 × 20 s; 4 × 40 s	6 min

### Recommendations specific to each radiotracer

#### [^15^O]water

**Fig. 6:**
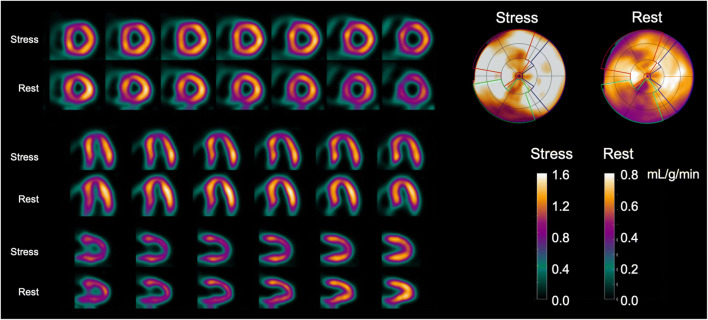
[^15^O]water parametric MBF images from a 65-year-old female with angina referred for assessment of ischaemia with PET. The images shown here are parametric MBF images based on 4-min dynamic [^15^O]water PET scans, with their corresponding polar maps. Note that colour scales of all images represent MBF in mL/g/min as seen in the colour bars. SPECT was negative. PET clearly shows balanced ischaemia with stress MBF far below the threshold of 2.3 mL/g/min

For [^15^O]water, no static images are available, but gating can be performed, and volumes and EF can be determined using first-pass blood volume images [16, 17]. For this, data has to be acquired in list mode so both dynamic (whole scan) and gated (circa 10–50 s post injection, depending on injection speed) images can be reconstructed. Visual interpretable images can be generated using digital subtraction techniques of blood volume from tissue as well as automated parametric images of MBF at the voxel level, but calculation of 3D perfusion images is preferred, and software packages capable of doing this are now becoming commercially available, whilst others can be obtained at no costs from academic centres that developed them. They now routinely generate 3D perfusion images (Fig. 6) as well as regional MBF and regional myocardial flow reserve (MFR, i.e. the ratio of stress and rest MBF) in absolute terms according to the standard 17-segment model of the AHA [66]. Although validated in large-scale clinical studies, there is currently no FDA approval for [^15^O]water use in medical practice, but its use is approved in many European countries.

#### ^82^Rb

At the end of the acquisition, the position of the heart on PET and CT acquisitions is visually matched for attenuation correction using dedicated software. From list mode acquisitions, three different PET reconstructed datasets can be obtained for both rest and stress studies: static (non-gated) acquisitions are reconstructed with PET data acquired between 90–120 s and 5–8 min after the injection, once the blood signal is low; gated acquisitions, which are reconstructed from PET data acquired within the same time interval post injection, usually divided in 8 (the best choice to contain noise) or 16 bins (better curve); and dynamic PET acquisitions. Several time frame schemes have been proposed for dynamic PET acquisition (Table 3). In patients with LVEF < 30%, the reconstruction of static and gated acquisitions should be started only 150–180 s after the injection to reduce the level of residual blood signal and improve the contrast between the myocardium and the LV cavity.

#### [^13^N]NH_3_

List mode acquisition is the current preferred modality for [^13^N]NH_3_. Dynamic imaging for absolute quantification is currently performed with the re-binning into frames of increasing duration, starting with shorter frames for the input function and tracer uptake phases (first 2–3 min) and then with 30 s or 1-min frames (Table 3). Static images constructed with the data after tracer extraction are available for visual assessment and are usually obtained together with cardiac gating. The alternatives for gating are 8 bins or 16 bins (see above).

## Interpretation of myocardial perfusion PET

### Perfusion images

#### Perfusion images using extracted radiotracers (^82^Rb and [^13^N]NH_3_)

After attenuation correction and reconstruction, the myocardial images must be reoriented along the myocardial axis as usually performed for myocardial perfusion imaging. Currently, most vendors already offer cardiac processing software on their devices, mainly the same as used for SPECT MPI. In case of physiologically retained tracers that allow visualising the relative perfusion images, it is important to proceed with the analysis of the reoriented slices and check for the image quality. The normalisation and side-by-side display of the three reoriented slice sets (short axis, vertical and horizontal long axis) is performed as usual. Even in the setting of quantitative myocardial PET, it is necessary to perform (and to report) the analysis of the myocardial uptake in qualitative terms. Presently, most processing software packages also provide polar map displays of myocardial uptake and permit the comparison of the individual patient results with a reference database (or allow the user to create an own normal data base). It is therefore possible, and in case recommended, to integrate the quantitative measurement of MBF with the assessment of the relative tracer distribution and with the comparison with a normal reference. Visual semi-quantitative image analysis is performed on a regional basis, using 17 segments (AHA model), and each segment is scored using a 5-point scale ranging from 0 (normal perfusion), 1 (mildly reduced perfusion), 2 (moderately reduced perfusion), 3 (severely reduced perfusion), to 4 (absent perfusion). This yields a summed perfusion score for both stress and rest myocardial perfusion images. The reported cut-off values to discriminate abnormal from normal PET MPI are diverse. The most widely accepted threshold is to consider a summed stress score (SSS) ≥ 4 as abnormal [67]. However, more restrictive thresholds have been proposed as well. For instance, Hsiao et al. classified an SSS > 0 as abnormal [68]. Similarly, Dorbala et al. proposed to transform the scores in percentage and then to consider an SSS% > 1% as abnormal [69].

#### Gated studies

Most programs simultaneously reorient and analyse both the gated images and a summed static image. The same programs that are employed for SPECT MPI usually process the gated PET studies as well. Accordingly, LV volumes and LVEF can be derived and synchrony assessments can be determined. The visual display of the gated studies using a cine loop function permits to evaluate the regional LV wall motion. End-diastolic and end-systolic perfusion polar maps, together with motion and thickening polar maps, can be obtained. Since, as above mentioned, the programs that perform the assessment of LV function in gated PET do not differ from those already extensively used and verified for gated SPECT, no separate normality values have been established. However, dissimilarities among the various software packages have been reported and should be considered in case of comparison between studies performed in different centres [70]. For [^13^N]NH_3_, there are relatively few data in large populations to support the reliability of gated PET for clinical purposes, but there are no reasons to hypothesise a different behaviour as compared with ^82^Rb-gated PET. Moreover, good agreement between [^13^N]NH_3_ and magnetic resonance imaging (MRI) for the assessment of LVEF, volumes and wall motion has been reported [71]. The side-by-side display of rest and stress gated images is useful to identify changes in LV wall motion and global function. With regard to the assessment of LVEF reserve (i.e. change in LVEF from rest to stress), it has been demonstrated that a LVEF reserve >+ 5% units has a very high negative predictive value for ruling out severe CAD, and conversely, a reserve <− 5% units has a very high positive predictive value for severe CAD [72]. Other reports confirm that a LVEF reserve < 0 is most probably related to abnormal perfusion and more severe CAD [67, 68]. It must be remembered that in the [^13^N]NH_3_ protocol, there is a slightly longer delay between stress and gated PET acquisition than when ^82^Rb is used. Thus, with [^13^N]NH_3_, it would be more correct to define the stress acquisition as an early post-stress one. On the other hand, the time interval is still far shorter than in gated SPECT, and there are data suggesting that indeed even with [^13^N]NH_3_, stress-induced changes on LV function can be identified [61, 73, 74].

#### Recommendations specific for [^15^O]water

As already reported, [^15^O]water perfusion PET provides routine quantification and studies have shown that grading of flow values outperforms the diagnostic accuracy of visual defect grading. It is, therefore, uncommon to express [^15^O]water PET in traditional terms of SSS, summed rest score (SRS) and summed difference score (SDS). The parametric images are used to identify visually the ischaemic area and its extent, whereby validated thresholds of absolute hyperaemic MBF and MFR are used to distinguish normal from abnormal perfusion. The most employed approach is based on the threshold formerly identified using receiver-operator characteristic analysis and recently validated in the PACIFIC study and classifies as abnormal the finding of at least two adjacent myocardial segments with hyperaemic flow of 2.30 mL/min/g or less [75, 76]. A similar method had been effectively used in the quantitative PET sub-study of the EVINCI trial [77]. The standard evaluation of gated PET based on uptake images cannot be performed with [^15^O]water, but LVEF can be determined using gated first-pass blood volume images covering the first minute after injection or gated parametric blood volume images [16, 17].

### Quantification of myocardial blood flow

#### General principles

**Fig. 7:**
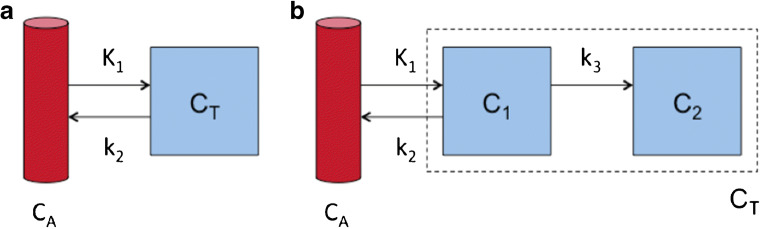
Compartment models: **a** single-tissue compartment model; **b** irreversible two-tissue compartment model. *C*_A_ is the radioactivity concentration in arterial blood, *C*_T_ the radioactivity concentration in tissue, with *C*_1_ and *C*_2_ describing free and internalised tracer in tissue, and *K*_1_, *k*_2_ and *k*_3_ are rate constants describing the transport rates of tracer between the different compartments

Compartment models can describe the kinetics of PET tracers for MBF. Figure 7, for example, shows a single-tissue compartment model, with rate constants *K*_1_, describing the transport rate in mL per g tissue per min from blood to tissue, and *k*_2_, which is the clearance rate from tissue per minute.

This simple compartment model can describe the kinetics of both [^15^O]water and ^82^Rb during the first minutes after injection. To accurately describe the kinetics of [^13^N]NH_3_, a second irreversible compartment should be added, with a rate constant *k*_3_ describing diffusion into the glutamine pool (Fig. 7). However, it has been proposed to disregard this compartment by limiting the analysis to the first 4 min and, thus, before that metabolic process begins [39]. For [^15^O]water, *K*_1_ is identical to MBF, and *k*_2_ is also proportional to MBF since water is freely diffusible and water that enters the tissue must be compensated by a similar amount clearing from the tissue to ensure mass balance. For ^82^Rb and [^13^N]NH_3_, the relationship between *K*_1_ and MBF can be described by the non-linear curves shown in Fig. 2, which have been established by comparison to either [^15^O]water or to animal studies with microspheres.

The equation describing the compartment model in Fig. 7a is as follows:

1 $$ {C}_{\mathrm{T}}(t)={K}_1{C}_{\mathrm{A}}(t)\otimes {e}^{-{k}_2t} $$

Here, *C*_A_(*t*) is the radioactivity concentration in arterial blood over time during the scan, and *C*_T_(*t*) is the radioactivity concentration in tissue over time. Since the radioactivity concentration measured within a volume of interest (VOI) or voxel in the myocardial wall also contains radioactivity in blood, as well as spill-over from the left- and right-ventricular (RV) cavities due to cardiac motion and the limited spatial resolution of PET, the total PET signal *C*_PET_(*t*) can be described as follows:

2 $$ {C}_{\mathrm{PET}}(t)=\left(1-{V}_{\mathrm{LV}}-{V}_{\mathrm{RV}}\right){K}_1{C}_{\mathrm{A}}(t)\otimes {e}^{-{k}_2t}+{V}_{\mathrm{LV}}{C}_{\mathrm{A}}(t)+{V}_{\mathrm{RV}}{C}_{\mathrm{RV}}(t) $$

where *V* = volume fraction. By fitting Eq. (2) to the measured time–activity curve in a volume or voxel, *K*_1_, *k*_2_, *V*_LV_ and *V*_RV_ can be estimated. Observe that the term before *K*_1_ in principle should be one minus the fractional blood volume, but since fractional blood volume and LV spill-over cannot be estimated separately, *V*_LV_, which is the sum of both, has to be used. This term is sometimes omitted.

#### [^15^O]water

The single compartment model to utilise [^15^O]water PET for quantification of perfusion has been validated over decades ago and remains the reference standard owing to the kinetic properties of this tracer that meet virtually all the criteria as a perfect flow tracer. The model incorporates partial volume correction as well as RV and LV blood pool spill-over effects. In the special case of [^15^O]water, *k*_2_ equals MBF/*V*_T_, where *V*_T_ is the partition coefficient of water in tissue, established to be 0.91 g/cm^3^ in myocardial tissue. In that case, MBF appears twice in Eq. (1), since *K*_1_ equals MBF. Hence, for [^15^O]water, Eq. (2) is generally re-written as:

3 $$ {C}_{\mathrm{PET}}(t)=\mathrm{PTF}\times \mathrm{MBF}\times {C}_{\mathrm{A}}(t)\otimes {e}^{-\frac{\mathrm{MBF}}{0.91}t}+{V}_{\mathrm{LV}}{C}_{\mathrm{A}}(t)+{V}_{\mathrm{RV}}{C}_{\mathrm{RV}}(t) $$

The PTF accounts for both underestimation of radioactivity concentrations due to spill-out effects, but also to overestimation due to spill-in, providing an intrinsic correction for partial volume effects which is exclusive to [^15^O]water. In addition, it can be seen as a measure for discrepancies between the influx and clearance rate of [^15^O]water caused by non-perfusable tissue, such as scar tissue, within the VOI or voxel. Thus, for [^15^O]water, MBF is measured only in perfusable tissue, in contrast to other PET tracers, which measure transmural MBF. Transmural MBF can then be computed indirectly as MBF × PTF/(1 + *V*_LV_ + *V*_RV_). A basis function implementation of Eq. (3) allows for fast calculation of MBF values at the voxel level, resulting in parametric images of MBF [78]. Dividing PTF with the anatomical tissue fraction (ATF) allows for calculation of the PTI, a marker of myocardial tissue viability [22, 23]. When the PTI concept was first introduced, ATF was estimated by subtracting a normalised blood volume image based on an additional C^15^O scan from a PET attenuation image [23], but using a modern PET/CT scanner, the blood volume image can instead be obtained from *V*_LV_ and *V*_RV_ in Eq. (3), and hence, using [^15^O]water, both MBF and viability can now be measured using a single scan [79]. Although so far mainly used in selected research centres, [^15^O]water has been quite extensively applied in the clinical setting, and reference values for identifying MBF and MFR abnormalities have been reported. In particular, the demonstrated effectiveness of the hyperaemic MBF for characterising CAD patients has been advantageous in the setting of hybrid imaging and of stress only quantitative PET. There are limited data about the comparison of [^15^O]water results achieved using different platforms. In the single available study, two non-commercial platforms gave tightly comparable results, with no significant differences at rest, and small variance under stress, but with a very good interclass correlation coefficient [80].

#### ^82^Rb

The use of ^82^Rb made the wide application of PET for MPI possible. However, already at the beginning of the 1990s, the advantages of performing quantitative PET were acknowledged. In particular, the group of Gould et al. developed a simplified approach to MBF quantitation, referred as the retention model, based on the acquisition of a 2-min image after tracer injection [81]. Although based on several assumptions, this method was effective in deriving useful MBF and MFR estimates, which could be integrated in the more comprehensive concept of coronary flow capacity [82]. More recently, compartmental models have been developed and validated [62, 63, 83]. Because of the wide use of this tracer, particular emphasis has been given to the reproducibility of the calculated values. With regard to this point, the one-tissue compartment model proposed by Lortie et al. has been demonstrated to be the most reproducible, even if implemented in different software platforms [64, 84–86]. However, there are differences in the measured values in normal subjects, which justify some caution in the comparison of data obtained using different platforms [87]. The approach used for defining the input function can affect the result agreement between different methods [88]. Using the prognostic implications as reference, MFR seems more consistent than hyperaemic MBF [88].

#### [^13^N]NH_3_

The approaches of MBF quantitation using [^13^N]NH_3_ are mainly based on compartmental models, although simplified approaches based on tracer retention have been used as well. In the retention approach, the signal in the myocardial wall is related to the net retention rate *K*_*i*_ multiplied by the integral of the plasma activity over time [2, 4].

4 $$ {C}_{\mathrm{PET}}(t)={K}_i{\int}_0^t{C}_{\mathrm{A}}\left(\tau \right)\mathrm{d}\tau $$

Although *K*_*i*_ underestimates MBF more than *K*_1_ does, it is still proportional to MBF as seen in the red curve in Fig. 2. The proposed compartment models are the two-compartment model, similar to the already mentioned one-tissue compartment model, and the three-compartment model, which takes into account the metabolism of [^13^N]NH_3_ after myocardial uptake (Fig. 7b) [4, 38, 40, 89, 90]. The latter model has been also simplified by limiting the analysis to the first 4 min after tracer injection and accordingly neglecting the metabolic fate of [^13^N]NH_3_ [39]. The results achieved by these different compartmental models have been regarded as to be closely related to each other, but nevertheless show significant differences [91]. The analysis has been more recently expanded to the combination of the compartmental model and software platforms, showing, together with the good general agreement between the measures, the presence of inter-modality variations in MBF, which are also partly influenced by patient characteristics [92, 93].

## Pitfalls and artefacts

### General considerations

A first and, unfortunately, unavoidable problem of quantitative PET is the impossibility to perform a physiological stress such as dynamic exercise to explore the MFR. As already mentioned above, this limitation is related to the need to obtain dynamic images during tracer injection and cannot be avoided even if perfusion tracers with longer half-life such as [^18^F]flurpiridaz will become clinically available. Whether a simplified approach based on the [^18^F]flurpiridaz standardised uptake values only will be effective for MFR assessment remains uncertain [94]. In addition, the need to place the patient in a supine position during acquisition is another potential problem of quantitative PET.

The problem of achieving a correct alignment between PET and CT images for attenuation correction is common to all types of cardiac PET investigation but can be particularly challenging in case of quantitative studies [95]. Moreover, in case of quantitative studies, there is the possibility of motion artefacts during the acquisition of the input function, which requires processing software with the capability of single frame realignment [57, 96]. This is probably one of the biggest technical challenges to quantitative cardiac PET. Particularly difficult to prevent and to correct is the presence of ‘myocardial creep’ due to pharmacologic stress [97]. Another major issue in quantitative PET is the quality of the injected bolus, which should show a single peak without evidence of detector saturation [98]. The time–activity curve should be examined to identify these possible interfering issues including a delayed start of the acquisition [98]. Moreover, patient motion can be detected as an abnormal hump in the later phases of the myocardial time–activity curve [98]. A general pitfall with the use of retention tracers ^82^Rb and [^13^N]NH_3_ is that input function and myocardial wall delineation are performed on the late uptake images and then transferred to the early dynamic images. This approach may introduce artefacts, especially in case of ‘myocardial creep’, as VOIs may differ between the early part and the later part of the acquisition. For [^15^O]water, where there are no uptake images, VOIs need to be defined on the dynamic data itself and as such [^15^O]water is less sensitive to ‘myocardial creep’–related artefacts. In any case, an effective means of identifying patient motion is to look at VOI placement over the myocardium during the course of the scan.

Independently of the above-mentioned potential pitfalls, the interpretation of the quantitative data might be hampered by a series of conditions, in which an abnormal peak MBF or (more frequently) an abnormal MFR is not directly an expression of myocardial ischaemia caused by epicardial CAD. In particular, patients with prior coronary artery bypass grafting (CABG) may have abnormal MBF in spite of patent grafts, although a trend towards normalisation after several months post-intervention has been described [99, 100]. Similarly, patients with LV dysfunction or severe kidney disease may have abnormal values without epicardial disease and with visual normal perfusion findings [101]. Conversely, patients with scar tissue due to prior myocardial infarction may present with abnormal low resting MBF causing false normal MFR values even if peak MBF is abnormal. In all these circumstances, the quantitative PET data must be cautiously evaluated. Image count density directly affects the diagnostic quality and reliability of the study. It is therefore important to realise that additional factors, such as body habitus and weight, radionuclide dose, scanner performance and acquisition time, also influence the final count density of PET images.

### [^15^O]water

Pitfalls of [^15^O]water are related to bolus delivery, PET resolution, relative count rate statistics, intermediate range of the positron, patient motion and suboptimal hyperaemia. For [^15^O]water, where there are no uptake images, VOIs need to be defined on the dynamic data itself, and as such, [^15^O]water is less sensitive to ‘myocardial creep’–related artefacts. In addition to the lack of uptake images, the main limitations for the analysis of [^15^O]water are the need to correct for the high activity in the blood pool and for the spill-over from LV and RV [102]. This correction is usually performed in the kinetic modelling and implemented in all available dedicated software packages that can process also [^15^O]water data. Specific commercial quantitative software programs for [^15^O]water PET have not been so far available, requiring in-house knowledge of kinetic modelling for analysis. However, some packages are currently becoming commercially obtainable. Measurement of LVEF based on [^15^O]water can be done using first-pass images, but this has not been extensively validated at this point [16, 17]. [^15^O]water PET MPI is clinically approved in Europe and reimbursed in several European countries. However, in the USA, [^15^O]water PET MPI has not been approved by the FDA for clinical use and is not reimbursed by third-party payers.

### ^82^Rb

The specific pitfalls of ^82^Rb imaging are related to the possibility of detector saturation during the tracer first pass and to the flattening of the blood first-pass curve caused by the decrease in activity of ^82^Rb per volume with ‘ageing’ of the generator. Regarding the first point, optimised injection protocols taking into account the characteristics of the state-of-the-art scanners have been proposed, which offer a good compromise between the risk of detector saturation during the first-pass phase and the need of adequate activity in later frames [103, 104]. Regarding the second point, novel injectors with the capability to deliver constant activity infusion rates are now available [105]. Another peculiar problem of ^82^Rb is the interference of the concurring prompt gamma emission, which happens in 13% of the decay events and requires a dedicated correction, with demonstrated favourable effects on image quality [106, 107]. Finally, it must be remembered that the high energy ^82^Rb positrons have the longest positron range among the myocardial PET tracers [20, 21, 108]. The lower extraction at high flow values could decrease the detectable difference between normally perfused and slightly hypoperfused myocardium under stress [108]. Abnormal tracer uptake in the lungs can be registered in patients with chronic obstructive pulmonary disease and in patients with LV dysfunction [109]. Gastric ^82^Rb uptake and spill-over can be seen in up to 10% of patients and affect the analysis of the inferior wall on PET MPI [110]. Proton pump inhibitor therapy seems to be associated with an increase in the intensity of the gastric ^82^Rb activity [110]. In addition, obesity and large stomach volumes were associated with more severe impact of the gastric signal on the PET MPI analysis, suggesting that appropriate fasting prior to ^82^Rb PET MPI may help to improve image quality, in particular in obese patients [111].

### [^13^N]NH_3_

In addition to the general pitfalls of PET MPI, [^13^N]NH_3_ has some specific issues. In particular, the regional uptake can be relatively reduced in the lateral wall, mainly in subjects with normal LV function [112]. More recent studies suggest that a major role in this finding is played by attenuation artefacts related to respiratory movements [113]. Apical thinning has been reported in PET MPI in general, but it is more pronounced with [^13^N]NH_3_ and in TOF cameras [114, 115]. As with ^82^Rb, abnormal tracer uptake in the lungs can be observed in patients with chronic obstructive pulmonary disease and in patients with LV dysfunction. Moreover, increased pulmonary tracer uptake has been described in heavy smokers as well [116]. As for all other perfusion tracers (PET as well as SPECT), abnormal visualisation of the RV wall can be observed in case of hypertrophy or because of chronic obstructive lung disease. If abnormal uptake in the RV wall is observed only under stress, it can be caused by a relatively lower uptake in the left myocardium related to severe CAD with diffuse ischaemia. Specific pitfalls for quantitative perfusion measurements can be related to the misalignment of emission and CT images for attenuation correction and to motion artefacts during the dynamic acquisition. On the other hand, the relatively lower activity using [^13^N]NH_3_ as compared with ^82^Rb reduces the risk of overflow and dead time losses during the first phases of bolus transit through the heart, and the higher quality of the uptake images with [^13^N]NH_3_ facilitates the definition of the VOIs needed for quantitative measurements. Because of the more prolonged half time, [^13^N]NH_3_ protocols are more time demanding. Performing the resting studies of more than one patient and then returning to the first patient for the stress part can overcome this limitation. However, although this approach improves the patient throughput, it has the drawback of requiring placing each patient two times separately on the examination bed and increases the problems related to patient positioning and to CT alignment. Thus, shortened protocols with correction of residual activity have been proposed [48, 49]. For gated PET assessment, the differences in timing of the stress study using [^13^N]NH_3_ protocols must be taken into consideration.

## Clinical evidence

### [^15^O]water

Beyond its characteristics of ideal tracer for MBF quantitation, [^15^O]water PET has been thoroughly validated for the diagnosis of CAD. Because in the early days visual parametric images were difficult to obtain, the majority of the validation studies focused on optimal thresholds for hyperaemic MBF and MFR to differentiate between healthy persons and patients with CAD (Table 4). Normal values of [^15^O]water PET MPI have been studied, including the influence of different subject characteristics on normal MBF (and MFR) [125, 126]. [^15^O]water PET MPI with a hyperaemic MBF cut-off at 2.5 mL/min/g showed a 92% diagnostic accuracy for the detection of CAD using invasive coronary angiography (ICA) and fractional flow reserve (FFR) as reference [59]. In addition, hyperaemic MBF (cut-off 1.86 mL/min/g) was more accurate (84%) than MFR (cut-off 2.3, 74%) for diagnosing CAD [60]. This superiority of hyperaemic MBF, although with slightly different thresholds, was confirmed in subsequent reports [75, 127]. In these studies, the optimal cut-off value to detect FFR assessed obstructive CAD ranged between 2.3 and 2.4 mL/min/g for hyperaemic MBF and was 2.5 for MFR [75, 117, 127]. The higher diagnostic accuracy of hyperaemic MBF as compared with MFR paved the way for stress only protocols. In addition, the PACIFIC trial revealed that [^15^O]water PET had higher diagnostic accuracy than SPECT MPI or CCTA [76]. It is important to note that such prospective studies are currently lacking for other PET perfusion tracers and no head-to-head comparisons are currently available. In addition, it should be kept in mind that these results were obtained in patients suspected of CAD but without a previous history of CAD and with a normal LVEF. Studies on other patient populations such as the PACIFIC II are currently ongoing. In analogy to the mounting body of evidence in other PET perfusion tracers, [^15^O]water PET also holds strong prognostic information and is incremental to anatomical coronary abnormalities as documented by CCTA [128].

**Table 4:** Proposed stress MBF and MFR threshold values for CAD detection

Reference	Tracer	Stress MBF cut-off	MFR cut-off
Kajander S et al. [59]	[^15^O]water	2.5 mL/min/g	
Danad I et al. [60]	[^15^O]water	1.86 mL/min/g	2.3
Danad I et al. [117]	[^15^O]water	2.2 mL/min/g	2.5
Danad I et al. [75]	[^15^O]water	2.3 mL/min/g	2.5
Hajjiri MM et al. [118]	[^13^N]NH_3_	1.85 mL/min/g	2
Fiechter M et al. [119]	[^13^N]NH_3_		2
Morton G et al. [120]	[^13^N]NH_3_		1.44
Anagnostopoulos C et al. [121]	^82^Rb	1.7 mL/min/g	2
Naya M et al. [122]	^82^Rb		2
Naya M et al. [123]	^82^Rb		1.93
Ziadi MC et al. [124]	^82^Rb		2

### ^82^Rb

Sensitivity and specificity for detecting an angiographically significant coronary stenosis of > 50% are 87% and 73% for SPECT MPI compared with 91% and 89% for ^82^Rb MPI, respectively [129, 130]. In a systematic review and meta-analysis evaluating the accuracy of ^82^Rb MPI in comparison with SPECT MPI for the diagnosis of obstructive CAD, fifteen ^82^Rb PET and eight cardiac SPECT studies were included [131]. ^82^Rb PET demonstrated sensitivity and specificity of 90% and 88% for the detection of obstructive CAD on ICA, whereas the sensitivity and specificity for ^99m^Tc-labelled tracer SPECT with ECG-gating and attenuation correction were 85% and 85%, respectively [131]. When patients with low prevalence of CAD were excluded, diagnostic accuracy was higher with ^82^Rb MPI than with SPECT MPI (area under the curve (AUC) 0.95 vs. 0.90; *p* < 0.0001) with a marked decrease in the specificity of SPECT MPI (70%) [131]. Three studies compared directly ^82^Rb MPI with SPECT MPI and found superior accuracy of ^82^Rb MPI [32, 132, 133]. Moreover, ^82^Rb MPI allowed for an effective prognostic patient risk stratification independently of the results of SPECT MPI [134]. A recent prospective study in women and obese patients confirmed the superior sensitivity of ^82^Rb MPI compared with SPECT MPI even if acquired with a cadmium zinc telluride camera—85% vs. 57%, *p* < 0.05 [135]. The addition of MBF quantification to the interpretation of MPI has proven clinically relevant, especially for the identification of patients with balanced myocardial ischaemia [121, 136, 137] (Table 4). Moreover, impaired MFR is associated with a worse prognosis in symptomatic patients with a visual normal PET MPI [122]. A value of global MFR > 1.9 excludes high-risk CAD with a negative predictive value of 97% [123]. Conversely, the prevalence of multivessel disease is high in patients with global MFR < 1.5 and intermediate for global MFR values between 1.5 and 2.0 with some overlap with microvascular disease [138]. In patients with an intermediate risk of mortality based on MPI, the addition of MFR allowed for the re-classification of 17% of patients into the high-risk group and 34% of patients into the low-risk group [138]. In addition, the decrease in global MFR values measured in 677 patients with ^82^Rb PET had a strong and incremental prognostic value over the extent of myocardial ischaemia [124]. In patients with known or suspected CAD, a multicentre observational study showed that the extent and severity of ischaemia and scar on PET MPI provided incremental risk estimates of cardiac death and all-cause death compared with traditional coronary risk factors [139]. In contrast to cardiac SPECT, images are acquired with ^82^Rb PET during the pharmacological stress. Subjects without CAD exhibit a rise in LVEF during pharmacological adenosine stress, whereas the absence of increase or even a drop in LVEF is associated with multivessel disease on ICA [72].

### [^13^N]NH_3_

The clinical value of [^13^N]NH_3_ PET has been well demonstrated. Early studies based on visual and semi-quantitative analysis had already demonstrated the superiority over ^201^Tl MPI in detecting myocardial ischaemia, and these results were confirmed using ^99m^Tc-labelled tracers [37, 140]. Subsequent studies including quantitative perfusion data showed an excellent diagnostic performance of the measurements for CAD detection and indicated that MBF and MFR have an added value over the visual assessment of perfusion [141]. As a further demonstration of quantitative [^13^N]NH_3_ PET reliability in terms of test–re-test variation, several studies showed that this approach can detect signs of asymptomatic CAD in subjects with elevated risk profile and then recognise MBF improvement after that an effective control of the risk factors had been obtained [142–148]. More recent studies indicated the added value of quantitative [^13^N]NH_3_ PET for characterisation of multivessel CAD [118, 119]. With regard to the prognostic implications of MBF and MFR measurements using [^13^N]NH_3_ PET, it was observed that these parameters were able to improve the prognostic stratification of subjects already classified according to their risk profile [149]. In a comparison between perfusion pattern and quantitative data, the adverse prognostic meaning of abnormal perfusion was confirmed, but a low MFR identified patients at risk of cardiac events even in case of normal perfusion pattern [150]. Similarly, the decrease in MFR was found to be a more sensitive predictor for cardiac death than LVEF, both in a general population of chronic CAD patients and in a cohort submitted to PET-driven revascularisation [151, 152]. Various studies have tried to assess the best thresholds to differentiate between normal and abnormal MBF and MFR in [^13^N]NH_3_ PET (Table 4). In a direct comparison between hyperaemic MBF (cut-off 1.52 mL/min/g) and MFR (cut-off 2.74), MFR showed a diagnostic superiority for detecting a significant coronary stenosis [141]. Subsequent studies indicated a threshold of 1.85 mL/min/g for hyperaemic MBF and of 2.0 for MFR [118]. In this last report, however, maximal MBF appeared more effective for detecting CAD than MFR [118]. On the other hand, the MFR cut-off of 2.0 was later confirmed [119]. In other studies, using a different quantification method, the MFR threshold was set at a lower level (1.4) [120].

## Clinical indications

The value of quantitative PET for CAD diagnosis and prognosis has been extensively validated [153–155]. However, as in general for PET studies as compared with their SPECT equivalents, costs and logistic problems, including the current prevalent use of PET scanners for oncology indications, make it necessary to identify specific patient subsets who can mostly benefit from PET MPI.

### Diagnosis

For diagnostic purposes, the most widely accepted indication for using quantitative PET is the suspicion of diffuse CAD, with the possibility of balanced ischaemia that could be missed by the assessment of relative tracer uptake. More in general, the added value of quantitative PET is accepted for patients with known CAD, in whom a more in-depth pathophysiological assessment of the disease is required, or in whom complex, multivessel disease is suspected [156]. Conversely, quantitative PET is useful in all patients with symptoms suggestive of myocardial ischaemia, in whom ICA does not show significant lesions, in order to identify microvascular disease [137]. This is particularly relevant in female patients [157, 158]. Given the increasing use of CCTA, there is a growing number of patients in whom the detection of borderline lesions requires further assessment of possible ischaemic burden. Patients with high body mass index represent another accepted indication for using PET because image quality is improved due to the high energy photons, high signal of PET tracers and accurate attenuation correction of PET [159]. Because of the more favourable dosimetric profile, PET should be preferred in young patients, particularly in young women.

### Prognosis

Several studies have demonstrated that quantification of MBF and MFR can be valuable for risk stratification [122–124, 138, 149–152, 160, 161]. In this regard, there is evidence that quantitative PET, putting together several important prognostic indicators, such as the extent of regional uptake defects, peak MBF, MFR and LVEF reserve, could be very useful as a tool for stratifying risk in CAD patients [162]. However, the choice of using PET is affected by the above-mentioned logistic and economic limitations, taking into account that gated SPECT MPI allows as well for good risk stratification in patients with chest pain. On the other hand, in patients with other conditions that may affect the coronary circulation, such as the cardiomyopathies, and above all hypertrophic cardiomyopathy, quantitative PET with its unique capability to assess the severity of microvascular disease is probably the most effective method to achieve an effective risk stratification [163–166]. Furthermore, quantitative PET is able to improve the prognostic stratification of groups already at increased risk, such as diabetics and patients with end-stage renal disease, and even in patients with chest pain and normal coronary arteries, due to microvascular disease [167–170]. Finally, quantitative PET is the most effective way to identify vasculopathy in heart transplant patients [171].

### Treatment management

Because of its capability to differentiate between the most severe stenosis and the other lesions, without missing even relatively limited reductions in coronary reserve, quantitative perfusion PET could be a very useful tool for guiding patient management, particularly in subjects with complex CAD [172, 173]. Single-site experiences suggest even a cost-effective capability to orient patient management [174]. Unfortunately, there are no randomised studies that assess the advantages of a PET-guided treatment strategy. Previous single-centre studies have demonstrated that quantitative PET data is able to identify asymptomatic CAD and to assess the results of therapy for risk factor management in these subjects [142, 143, 145, 146, 148]. More recently, differences in hyperaemic MBF and MFR between patients with resistant hypertension and those effectively treated have been demonstrated [65].

## Recommendations for PET MPI reports

**Fig. 8:**
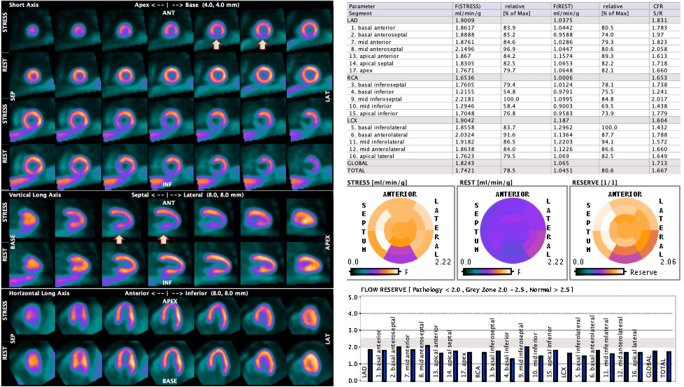
[^13^N]NH_3_ PET of a patient with three-vessel disease. The uptake images (left panel) show a stress-induced inferior wall perfusion defect (arrows), which is confirmed by quantitative PET analysis (right panel), demonstrating clearly reduced stress MBF and decreased coronary flow reserve (CFR = MFR) in the right coronary artery territory (RCA). However, mildly abnormal stress MBF and MFR are observed in the left anterior descending (LAD) and left circumflex (LCX) territories as well. In gated PET analysis, LVEF decreased from 54% at rest to 48% after stress, confirming the presence of diffuse ischaemia

As expected, the construction of a correct report for a complex investigation such as quantitative cardiac PET is demanding. Table 5 summarises the items that should be included. Particular attention should be paid to a clear identification of the indication for the study, because this influences the emphasis that should be given to the several parameters that can be derived from the investigation. The accent on quantitative PET data does not make the value of the visual assessment of tracer uptake superfluous. The number of ischaemic or necrotic segments within the 17-segment AHA classification, the SRS, SSS and SDS scores and the percentage of ischaemic burden should therefore be detailed. Similarly, it is necessary to describe wall motion abnormalities and report rest and stress LVEF values. As for the quantitative PET data, it is important to describe with utmost accuracy the technical modalities of the study, including the employed software and model, since these affect the final measurements and should be considered in case of comparison with prior reports, especially if produced by another centre. As mentioned above and apart from [^15^O]water, there is no consensus on the thresholds for normal and abnormal hyperaemic MBF or MFR for the different tracers, and it would be desirable to include those adopted, possibly with the proper reference. In case of [^15^O]water studies, the part about the visual assessment of tracer uptake in terms of semi-quantitative scores and gated PET data could be omitted. On the other hand, various groups using [^15^O]water consider positive for CAD any patient with at least two adjacent segments within a coronary territory classified as abnormal using a validated threshold (hyperaemic MBF < 2.3 mL/g/min), if this finding is confirmed by a visual defect in the parametric perfusion images [75]. Centres using hybrid imaging will include the data related to calcium scoring and possibly even those related to CCTA (see infra, “Hybrid imaging” section). The final conclusion of the report should include an interpretation of all reported findings and, most importantly, their connection in order to answer the specific clinical question. A particularly difficult point is the differentiation between balanced three-vessel myocardial ischaemia and diffuse microvascular disease. Among the possible criteria, a homogeneous reduction of MBF without detectable perfusion defects is considered more indicative of microvascular disease, whilst a more heterogeneous reduction is regarded as suggestive of balanced ischaemia due to CAD (Fig. 8).

**Table 5:** Scheme for quantitative PET reporting

Administrative data	
• Hospital name, including department, address, contacts	
• Study identification number	
Patient-specific information	
• Patient identification, including personal data, sex, date of birth, height, weight, patient code and archive number	
• Relevant history, including risk factor profile, previous cardiac events, prior revascularisation procedures, symptoms and current medications	
• Indication for the study and specific clinical question to be answered by the investigation	
Study-related data	
• Type of study	
• Study date	
• Interpretation date	
• Radiopharmaceutical, injected activity at rest and at stress, type of stress agent	
• Acquisition protocol including description of dynamic parameters and framing of the gatedstudy	
• Rest blood pressure and heart rate	
• ECG at baseline	
• Peak stress blood pressure and heart rate	
• Presence of symptoms and ECG changes during the stress test	
• Quality assessment of the acquired images	
• Description of the processing software and compartmental model applied to the quantitative analysis	
Image reporting	
• Image description with visual analysis of resting and stress images, whenever available,with reference on the 17-segment model for territory identification	
• Scoring of the 17-segment model, with calculation of SRS, SSS and SDS (not for [^15^O]water)	
• Definition of the perfusion normality vs. abnormality according to the accepted criteria taking care to assign the perfusion defects to the related coronary territory, according to the standard distribution or to the patient coronary distribution pattern if known	
• Normal SSS = 0–3 (< 5% myocardium); mildly abnormal SSS = 4–7 (5–10% myocardium) moderately or severely abnormal SSS > 8 (> 10% myocardium) (not for [^15^O]water)	
• Visual estimate of LV dimensions and transient ischaemic dilation	
• Abnormal visualisation of the right ventricle and its possible enlargement	
• Extracardiac findings, such as abnormal lung uptake (not for [^15^O]water)	
Quantitative analysis	
• Resting MBF (corrected for the rate pressure product if the resting heart rate and/or the baseline blood pressure is abnormally elevated) with range of the segmental values and both the single territory values and the global left ventricular value	
• Stress MBF, described as above	
• MFR described as above	
• Summary of findings in term of segments/territories with peak MBF/MFR below the normal threshold (identified according to the tracer and the model used for data analysis)	
Gated PET acquisition	
• Resting LV volumes, EF and wall motion abnormalities, to be described qualitatively and scored with a proper point-scale in terms of motion and thickening according to the standard 17-segment scheme	
• Stress LV volumes, EF and wall motion abnormalities, to be described qualitatively and scored with a proper point-scale in terms of motion and thickening according to the standard 17-segment scheme	
• LVEF reserve	
CT (images of adequate quality)	
• Evaluation of coronary artery calcium scoring (description) and Agatston score; description of abnormal extracardiac findings on the CT	
Hybrid PET/CCTA	
• Correlation between MBF and the main findings of the CCTA (e.g. location of significant coronary obstructive disease and downstream MBF)	
Conclusion	
• Clinical interpretation of visual findings, MBF and MFR and gated PET data (whenever applicable)	
• Specific answer to the clinical question, and if needed recommendation for additional imaging

## Perspectives for PET MPI

### Hybrid imaging

Cardiac hybrid imaging offers the ability to combine strengths of different imaging modalities for anatomical and functional evaluation of CAD [175–177]. Hybrid images contain two datasets that are combined into a fused image in which both modalities contribute important information. This can be achieved with a hybrid scanner or with separate imaging systems by software-based image fusion. Hybrid scanners combining PET with a multidetector CT, which can perform CT assessment of coronary anatomy, are becoming the standard for almost all commercially available devices [175]. In addition to CT, the newest generation of scanners offers the combination of PET with MRI [177].

CCTA has become an alternative to diagnostic ICA for the evaluation of many patients with suspected CAD. It shows very high sensitivity (> 95%) for the detection of coronary artery stenosis as compared with ICA [178, 179] and allows exclusion of obstructive CAD with high confidence. However, CCTA is a morphologic imaging tool that does not provide information on the haemodynamic consequences of a coronary artery stenosis. Thus, hybrid imaging combining CCTA and MPI may assist in differentiating haemodynamically significant from non-significant stenosis. Furthermore, assessment of coronary atherosclerotic burden by CT may improve the diagnostic and prognostic value of MPI [180–183].

**Fig. 9:**
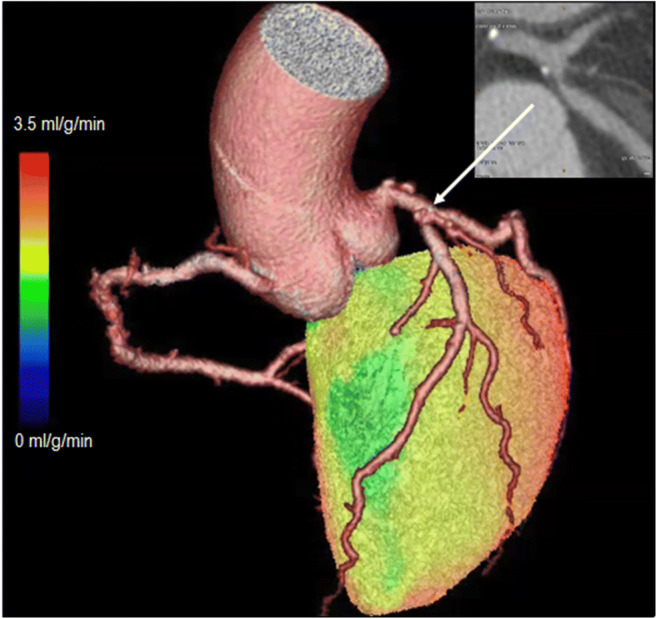
Hybrid PET/CT image demonstrating 3D reconstruction of coronary anatomy and MBF. CCTA shows a stenosis in the proximal left anterior descending coronary artery (insert), and [^15^O]water PET shows reduced MBF (green colour) in the myocardium subtended by the artery during adenosine stress

Hybrid imaging can provide accurate spatial alignment of CT and MPI data to improve co-localisation of myocardial perfusion abnormalities and subtending coronary arteries (Fig. 9). Hybrid imaging studies have shown that standard distribution of myocardial territories corresponds with the real anatomic coronary tree in only 50–60% of cases, which may cause misleading interpretation [184].

#### Hybrid imaging protocols

Hybrid imaging is a relatively novel technology and there is still a need to clarify which patients can benefit most from hybrid imaging, how to optimally combine different modalities and what is the cost-effectiveness of hybrid imaging [175–177]. MPI and CCTA can be performed in a single session with PET/CT scanners that have a minimum of 64 detector rows. Alternatively, a coronary calcium scan without contrast injection obtained by electron-beam computed tomography (EBCT) or CT can be combined with perfusion imaging. Sequential testing, perfusion imaging followed by CCTA or CCTA followed by perfusion imaging, in selected patients with uncertain or equivocal findings in the first test, is also an option. Differentiation of obstructive CAD and microvascular dysfunction is possible only if both CCTA and perfusion imaging are performed. Whilst CCTA is currently recommended as the initial diagnostic test primarily in patients with a lower range of pre-test probability of CAD, it can be assumed that patients at the higher range of pre-test probability or with known CAD are those that will most likely benefit from evaluation of myocardial ischaemia [185].

Patient preparation and imaging protocols for CCTA, coronary calcium imaging and MPI are mainly the same as for the individual scans [175, 176, 186]. Although β-blocker may reduce the extent and severity of perfusion abnormalities [187], one study indicates that diagnostic accuracy of PET perfusion imaging was maintained in patients who received i.v. β-blocker to slow down the heart rate before CCTA [188]. Proper indications and possible contraindications, such as contrast allergy, should be reviewed separately for PET and CT studies [176]. Analysis software that is able to handle fused images and data should be used in addition to independent analysis of anatomic and functional images. Dedicated cardiac fusion software packages are now commercially available allowing hybrid imaging with an excellent interobserver reproducibility and short processing durations [189, 190]. These software packages, following automated/semi-automated segmentation, generate 3D reconstructions of myocardial perfusion data that are fused with 3D coronary anatomy datasets resulting in a hybrid 3D visualisation. Verification of adequate co-registration and possible manual correction is needed to avoid misalignment, because of mismatches in the respiratory phases between the two datasets, minor beat-to-beat variations in the heart position or different ventricular size in ECG-gated and non-gated datasets [175, 176, 191].

#### Radiation exposure

Hybrid PET/CCTA imaging will increase the radiation dose to the patient since both techniques utilise ionising radiation. Using commonly available single-source 64-slice CT scanners with a prospectively ECG-triggered step-and-shoot acquisition protocol, it is possible to consistently perform a CCTA with an absorbed radiation dose of between 2 and 5 mSv [192]. Coronary calcium scan adds less radiation (approximately 1 mSv) to the patient than CCTA [175, 176]. Hybrid PET/MRI imaging can be performed with lower radiation exposure to the patient than PET/CT, which might be particularly beneficial in younger patients and/or in patients who may need repeated scans [177].

#### Clinical evidence

Validation studies have shown that cardiac hybrid PET/CT imaging is feasible and has high diagnostic accuracy in the detection of obstructive CAD [60, 126, 193–196]. A meta-analysis compared hybrid CCTA and perfusion imaging with SPECT, PET or MRI with CCTA alone (12 diagnostic studies and 951 patients in total) for the detection of CAD defined as luminal diameter reduction of at least 50% by ICA [197]. This meta-analysis found that pooled sensitivity of hybrid imaging was comparable to that of CCTA on per-patient (91% vs. 90%) and per-vessel (84% vs. 89%) basis. However, specificity of hybrid imaging clearly outperformed that of CCTA alone both on per-patient (93% vs. 66%) and on per-vessel (95% vs. 83%) analysis. There was also a modest improvement in overall diagnostic performance on per-vessel level (AUC 0.97 vs. 0.92). A limitation of the meta-analysis and current studies is that in many of them angiographic stenosis diameter is used as the reference standard instead of invasive FFR. The recent single-centre prospective PACIFIC trial compared hybrid imaging with stand-alone imaging in 208 patients who underwent CCTA, SPECT perfusion imaging and [^15^O]water PET perfusion imaging and ICA combined with measurement of FFR in all arteries [76]. In this study, the addition of functional imaging to CCTA improved specificity, but there was an increase in false negative findings that resulted in no overall incremental diagnostic benefit as compared with stand-alone imaging. However, studies comparing hybrid imaging with either stand-alone or side-by-side interpretation of the datasets have shown that interpretation was changed based on fused images in almost one-third of patients evaluated for suspected obstructive CAD [198–201]. Additional patient groups in which hybrid imaging has been shown to provide complementary information are those with congenital coronary anomalies [202] and patients with recurrent chest pain after CABG [203]. Furthermore, hybrid imaging can identify patients with microvascular dysfunction without obstructive CAD [204]. These studies indicate that the greatest added value of hybrid imaging is the exclusion of haemodynamic significance of coronary abnormalities seen on CCTA and the differentiation of epicardial and microvascular disease as well as correct localisation of the culprit lesion causing ischaemia.

The prognostic value of hybrid CCTA and SPECT MPI was demonstrated in a follow-up study in 324 patients [205]. A matched finding of myocardial ischaemia in a territory supplied by a stenotic coronary artery was associated with higher risk of death or myocardial infarction than unmatched ischaemia and stenosis. In another study, abnormal stress myocardial blood flow by [^15^O]water PET combined with stenosis on CCTA was associated with increased risk of death, myocardial infarction or unstable angina [206]. Revascularisation rates have also been highest in the presence of perfusion abnormality matched with a stenosis in the subtending coronary artery [207, 208].

Coronary artery calcium imaging can provide complementary information to MPI, because of its ability to quantify overall atherosclerotic burden [183, 209–213]. High atherosclerotic burden is associated with an increased likelihood of obstructive CAD [209, 211] and the risk of cardiac events in patients with normal myocardial perfusion scan [210], whereas a coronary artery calcium score of 0 identifies a low-risk patient population [183]. Coronary artery calcium score is inversely associated with both hyperaemic MBF and MFR providing incremental information over established CAD risk factors for predicting coronary vascular dysfunction [212, 213].

Hybrid PET/MR scanners have been available for a relatively short time due to technical challenges in this setup [177]. Different [^18^F]FDG administration protocols have shown potential for multimodality PET/MR evaluation of cardiac infiltration in multiorgan disease [214, 215], myocardial viability [216, 217] and inflammation [218, 219]. However, the experience from myocardial perfusion imaging with PET/MR scanners is limited [177]. The use of ^82^Rb for PET/MR protocols is difficult, since the ^82^Rb generator is not compatible with the MR setting. [^18^F]flurpiridaz (see below) with its longer half-life may be better suited for PET/MR studies to investigate the extent of myocardial infarcted area by MR and myocardial perfusion by PET.

### New radiotracers ([^18^F]flurpiridaz)

[^18^F]flurpiridaz is a novel PET MPI tracer labelled with ^18^F that binds to mitochondrial complex 1. Preclinical studies demonstrated [^18^F]flurpiridaz PET MPI to have rapid uptake in the myocardium, prolonged retention and superior extraction vs. flow profiles compared with nuclear tracers used in SPECT MPI [220–225]. Due to its favourable half-life (i.e. 110 min), [^18^F]flurpiridaz may be produced as a unit dose from a regional cyclotron, thus obviating the need for an on-site cyclotron or generator [35, 108]. Phase 1 [^18^F]flurpiridaz studies have shown that this tracer is clinically safe, has acceptable clinical dosimetry, and provides high-quality images in conjunction with exercise treadmill testing as well as pharmacological coronary vasodilation stress testing [226, 227]. Preliminary data suggested as well a potential value for quantitative assessments including MBF and MFR [94, 223].

A phase 2 study, BMS747158-201, consisting of 2 cohorts, was conducted to develop and subsequently to evaluate the diagnostic performance of 1-day rest/stress PET imaging protocols in patients with known or suspected CAD. An initial cohort was used to identify the appropriate activity and timing of [^18^F]flurpiridaz injection for a 1-day rest/stress protocol. In the second cohort, 143 patients presenting with a broad spectrum of pre-test likelihood of CAD were enrolled to evaluate the sensitivity, specificity and accuracy of [^18^F]flurpiridaz with the optimised 1-day rest/stress protocol; ICA or 3-month follow-up for cardiac events was used as the reference standard. Of the 143 subjects enrolled, 125 had an interpretable scan. PET MPI sensitivity was 76.9% for all readers, and specificity ranged from 74.0 to 87.7%. SPECT MPI sensitivity ranged from 59.6 to 71.2%, and specificity ranged from 76.7 to 89.0% [228].

The phase 3 study BMS747158-301 (ClinicalTrials.gov Identifier: NCT01347710) was an open-label, international multicentre study for the assessment of CAD using [^18^F]flurpiridaz PET MPI compared with SPECT MPI in patients with suspected or known CAD who were referred for ICA. Patients underwent a 1-day rest/stress protocol with ICA used as gold standard. The primary objective of this study was to assess the diagnostic efficacy (sensitivity and specificity) of [^18^F]flurpiridaz injection PET MPI compared with SPECT MPI. A total of 755 patients had evaluable rest and stress SPECT MPI and [^18^F]flurpiridaz PET MPI procedures as well as reference ICA. Although statistically significant superiority in sensitivity was demonstrated for [^18^F]flurpiridaz PET MPI over SPECT MPI across readers (*p* < 0.001), the endpoint of demonstrating the non-inferiority of [^18^F]flurpiridaz PET MPI to SPECT MPI in specificity was not met, with [^18^F]flurpiridaz PET MPI reaching a specificity of 76.2%, compared with SPECT MPI specificity of 86.8%. Thus, another phase III trial (AURORA, ClinicalTrials.gov Identifier: NCT03354273) is currently ongoing, with the purpose to get the final approval for the clinical use of [^18^F]flurpiridaz. However, a most recent retrospective evaluation on a cohort of the first phase 3 study has demonstrated that stress MBF quantified using [^18^F]flurpiridaz and the previously validated two-tissue compartment model are able to accurately identify CAD both on a per-patient and a per-vessel basis [94, 223, 229].
